# A randomized controlled trial of smoking cessation methods in patients newly-diagnosed with pulmonary tuberculosis

**DOI:** 10.1186/s12879-016-1727-4

**Published:** 2016-08-05

**Authors:** Mahshid Aryanpur, Mostafa Hosseini, Mohammad Reza Masjedi, Esmaeil Mortaz, Payam Tabarsi, Hamid Soori, Habib Emami, Gholamreza Heidari, Mehdi Kazempour Dizagie, Masoud Baikpour

**Affiliations:** 1 Tobacco Prevention and Control Research Center, National Research Institute of Tuberculosis and Lung Diseases (NRITLD), Shahid Beheshti University of Medical Sciences, Tehran, Iran; 2 Department of Epidemiology and Biostatistics, School of Public Health and Institute of Public Health Research, Tehran University of Medical Sciences, Tehran, Iran; 3 Shahid Beheshti University of Medical Sciences, Next to Ayatollah Taleghani Hospital, Evin, Tehran, Iran; 4 Department of Immunology, National Research Institute of Tuberculosis and Lung Diseases (NRITLD), Shahid Beheshti University of Medical Sciences, Tehran, Iran; 5 Division of Pharmacology and Pathophysiology Utrecht Institute for Pharmaceutical Sciences, Faculty of Sciences, Utrecht University, Utrecht, The Netherlands; 6 Clinical Tuberculosis and Epidemiology Research Center, National Research Institute of Tuberculosis and Lung Diseases (NRITLD), Shahid Beheshti University of Medical Sciences, Tehran, Iran; 7 Safety Promotion and Injury Prevention research center of Shahid Beheshti, University of Medical Sciences, Tehran, Iran; 8 Mycobacteriology Research Center, Biostatistics unit, National Research Institute of Tuberculosis and Lung Diseases (NRITLD), Shahid Beheshti University of Medical Sciences, Tehran, Iran

**Keywords:** Pulmonary tuberculosis, Smoking cessation, Intervention studies, Iran

## Abstract

**Background:**

Tuberculosis (TB) and tobacco use are two major alarming global health issues that tend to be co-prevalent in many developing countries and various surveys have provided evidence on their entangled associations. Accordingly, it is strongly suggested that smoking cessation be incorporated in TB control programs. Therefore, we aimed to evaluate the effectiveness of two smoking cessation methods among newly-diagnosed pulmonary TB patients.

**Methods:**

A total of 210 newly-diagnosed pulmonary TB patients from Tehran, Iran with smoking habits were included in this randomized clinical trial during 2012–2013. Patients were assigned to three groups of control (just TB medical treatment), brief advice (TB medical treatment plus individualized counseling sessions of quitting behavioral therapy) and combined intervention (TB medical treatment plus individualized counseling sessions of quitting behavioral therapy plus medical treatment with slow release bupropion). Patients’ abstinence was followed at six time point during six months. Data were analyzed by SPSS v.22 using Generalized Estimating Equations (GEE) model.

**Results:**

Abstinance rate at the end of six months were 71.7 % for combined intervention group, 33.9 % for brief advice group and 9.8 % for the control group (*p* < 0.001). Combined intervention group and brief advice group respectively had 35 times (*p* < 0.001, OR = 35.26, 95 % CI = 13.77–90.32) and 7 times (*p* < 0.001, OR = 7.14, 95 % CI = 2.72–18.72) more odds of not being an active smoker at each time point, compared to the control group.

**Conclusion:**

Considering the prevalence and importance of TB and the substantial influence of these preventive measures on controlling tobacco use, application of such programs is recommended.

**Trial registration:**

The survey was registered in the Iranian registry of clinical trials website (irct.ir) in August 31, 2013 with IRCT ID: IRCT2013062613783N1.

## Background

Tuberculosis (TB) and tobacco use are two major alarming global health issues posing immense threats to human populations [1, 2]. The annual mortalities related to these two epidemics are currently estimated to exceed seven million people [2]. TB and tobacco smoking tend to be co-prevalent and many developing countries are shouldering the concurrent burdens of the two outbreaks, simultaneously [3, 4]. Furthermore, various surveys have provided evidence on the relation between tobacco use and TB disease. Cigarette smoking is found to be associated not only with TB disease and tuberculous infection, but also with delayed bacteriologic clearance, increased susceptibility to infection, recurrence and TB related deaths [5–10]. The prevalence of smoking has also been found to be higher among TB patients compared to control groups and normal populations [11–15].

Tobacco smoking is associated with decreased proinflammatory cytokine release, decreased level of immunoglobulins in blood circulation and decreased CD4+ to CD8+ ratio causing suppression of both cell mediated and humoral mediated immunities that can lead to TB infection and improper treatment response [16–19]. The immunological changes are reported to be reversed within 6 weeks of quitting.

Although conventionally all the physicians suggest that their patients quit smoking, no separate counseling sessions are held specially for this important issue and most physicians just briefly mention the benefits of smoking cessation. Accordingly, it is strongly suggested that smoking cessation be incorporated in tobacco control programs [3]. Various behavioral methods have been proposed for smoking cessation in TB patients such as brief advice, which has been proven to considerably increase the rate of smoking cessation in these subjects [20]. International Union Against Tuberculosis and Lung Disease has proposed different methods of implementing brief advice, which have conformed to one concise easy method according to the national guidelines of United States of America [21]. Another method known as combined intervention has also been developed which embodies both simplified counseling models involving brief advice and pharmaceutical treatments [4, 14]. This method has also been found to be effective on smoking cessation of the evaluated subjects.

These simple interventions can provide vital information for TB patients on the necessity of prevention from exposure to tobacco smoke, without the need for complicated and costly measures. However, in spite of the urgent need, most specialists and medical staff are not familiar with these services [22].

Despite the available evidence on the effects of educational interventions on smoking cessation in TB patients [20, 21, 23–26], further investigations are required to find the best program for this means. Therefore, considering the correlation between smoking and TB disease and the importance of quitting in pulmonary TB patients, for the first time in Iran, this study aimed to evaluate the effectiveness of smoking cessation methods among newly-diagnosed pulmonary TB patients.

## Methods

This study was conducted during December 2012 to February 2014 on newly-diagnosed smear positive pulmonary TB patients with smoking habits to evaluate the effects of two smoking cessation methods. Eligibility regarding the patients’ smoking status was assessed according to self-declaration of subjects based on the guidelines of World Health Organization and International Union Against Tuberculosis and Lung Disease [5]. Inclusion criteria were as follows:

- Newly-diagnosed pulmonary TB patients according to a positive sputum smear (based on treatment guideline of WHO) [27]
- Patients classified as Category I (newly-diagnosed TB patients)
- Aged 18 years or older
- Persian speaking patients

Exclusion criteria included:

- Extra-pulmonary TB (brain, pericardium, adrenal glands, etc.)
- Multi drug resistance
- Co-infection with HIV/AIDS
- Opium addiction
- Patients classified as Category II (recurrence, treatment failure or treatment errors)
- Patients classified as Category III (chronic TB)
- Contraindications for treatment with bupropion
- Not willing to participate
- Unable to communicate and comprehend the written consent form

The study was designed on the basis of a homogeneous sample population and since following up patients with newly-diagnosed TB was more convenient, this group was chosen as the target population and patients with extra-pulmonary TB were excluded. Moreover, most HIV positive patients are IV drug abusers and are known to use various drugs; hence, inclusion of these patients might have led to inclusion of multiple confounding factors into our survey so we decided to exclude these patients as well. Patients with resistant TBs were also excluded due to their prolonged treatment course and long-term follow ups which were not compatible with this survey’s design. Category II and III patients were also excluded since their follow up trends were incompatible with our study’s protocols.

### Sampling

The city of Tehran (capital of Iran) was divided into three districts regarding the coverage of health centers by each of the three medical universities. Two health centers implementing the Directly Observed Treatment Short Course (DOTS) strategy were chosen from each district, according to the population covered by the centers and their consent for participation. 1530 TB patients referring to the six selected health centers or the referral hospital of Masih Daneshvari during December 2012 to February 2014 were evaluated and 210 newly-diagnosed pulmonary TB patients with positive smoking habits were enrolled in this study.

Using random permuted block numbers and stratifying according to age, gender and nationality, the included subjects were randomly assigned to three groups of combined intervention, brief advice and control.

### Data collection and interventions

In their first session all the patients included were evaluated regarding their tobacco use status, history of smoking, nicotine dependency (based on Fagerstrom test), reasons for smoking (based on WHO standard tests), the extent to which they were willing to quit (based on the trans-theoretical model [5]: pre-contemplation, contemplation, preparation, action and maintenance) and motivation for quitting.

The control group just received the DOTS regimen, while the other two groups participated in educational interventions additional to the DOTS course. During the first two weeks, the patients assigned to the brief advice group participated in 4 sessions of smoking cessation counseling based on behavioral therapy. The intervention was designed according to the manual of smoking cessation interventions for TB patients [26], through which necessary information about the benefits of quitting considering their underlying disease were explained clearly for the subjects.

The 5A’s protocol including Ask, Advise, Assess, Assist and Arrange [5] was utilized for the combined intervention group. In the first two weeks four counseling sessions were held giving each patient personalized consultations including behavioral therapy for quitting. The educational intervention was based on the manual of smoking cessation interventions for TB patients [26]. All the subjects were also treated by slow release bupropion (wellban ER, Abidi) given 150 mg/d in the first week followed by 300 mg/d till the end of the ninth week. All the medical treatments and counseling sessions for all the three groups of control, brief advice and combined intervention were delivered by one trained physician in each center.

Finally smoking cessation was evaluated in all three groups by assessing the expiratory carbon monoxide (CO) concentration via PICO Smokerlysers device (Bedfont Scientific Maidstone, UK) based on standard criteria [28]. Exhaled CO level was evaluated 6 times for each patient at the end of the second, third and fourth counseling sessions in the first two weeks, in the second month follow up session and at the end of the fourth and sixth month follow-ups. Patients were categorized into two group based on the level of their exhaled CO level at each time point in a way that patients with an exhaled CO level of less than 7 ppm were considered as nonsmokers and subjects with a CO level of higher than 7 ppm were categorized as smokers. These assessments were performed by a different physician in each center, blinded to the patients’ groups.

### Statistical analysis

The data were entered into SPSS (v.20) and checked for outliers. In order to check that the distribution of demographic and smoking-related characteristics were not statistically different in the study groups, Chi-squared test (e.g. for sex and marital status) and one way ANOVA (e.g. for smoking initiation age, quit motivation score) were used. Then, as the study groups were found homogeneous according to these factors, to evaluate the effects of smoking cessation methods in newly-diagnosed pulmonary tuberculosis patients, Generalized Estimating Equations (GEE) model with binary outcome was used to take the time differences into account. Moreover, various structures (unstructured, independent …) were evaluated for the correlation matrix and the model with the lowest Quasi-Akaike Information Criterion (QIC) and the best goodness-of-fit was applied as the final model.

### Ethical considerations

All the necessary information was thoroughly explained for the eligible patients and from the subjects willing to participate in the survey, an informed written consent was obtained. The study methods were approved by the Research Ethics Committee of Tuberculosis and Lung Disease research center and the Ethics Committee of Shahid Beheshti University of Medical Sciences. The survey was registered in the Iranian registry of clinical trials website (irct.ir) in August 31, 2013 with IRCT ID: IRCT2013062613783N1.

## Results

**Fig. 1:**
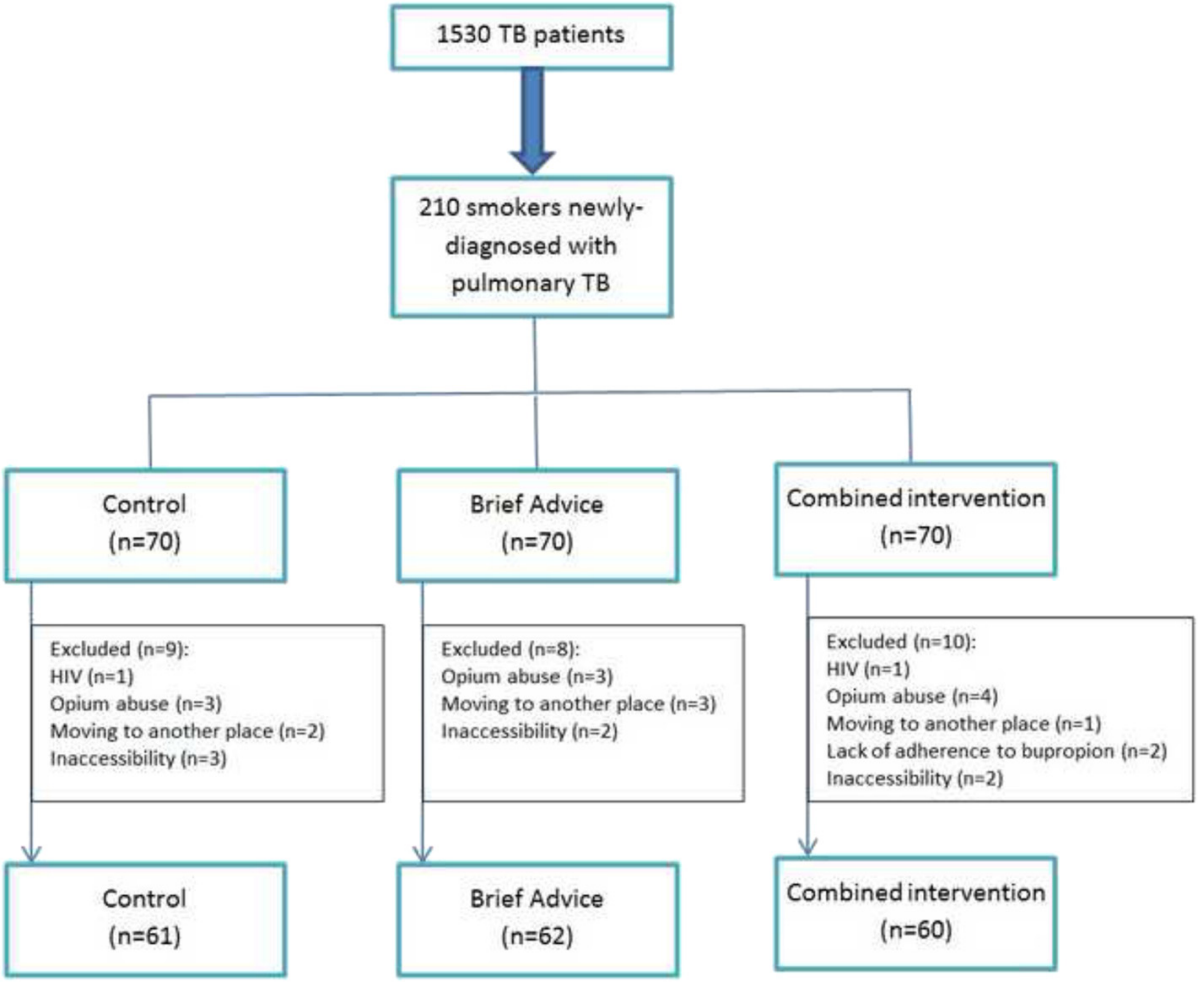
Details regarding total number of eligible patients and breakdown of reasons for exclusion

Two hundred and ten newly-diagnosed patients were found eligible to be included in the survey, of which 27 were excluded later due to a delayed positive HIV test, opium abuse, moving to another place to follow their treatment, lack of proper adherence to bupropion treatment and inaccessibility of the subject. Finally 60 patients were assigned to the combined intervention group, 62 subjects to the brief advice group and 61 patients to the control group (Fig. 1).

On allocation, subjects were stratified according to age for sex and nationality. However, statistical analyses were also performed on all the characteristics of the subjects including age, living area, education and occupation to ensure the randomization of allocation. The results were quite close to each other and no significant intergroup difference was found regarding demographic characteristics of the subjects (Table 1).

**Table 1:** Demographic characteristics of the subjects in each group recruited from Tehran, Iran during 2012–2013

Demographic factor		Control	Brief advice	Combine intervention	*p*-value
sex	Male	55 (90.2 %)	56 (90.3 %)	54 (90.0 %)	0.99
	Female	6 (9.7 %)	6 (9.7 %)	6 (10.0 %)	
Age		47.36 ± 16.30	45.53 ± 16.43	45.05 ± 15.81	0.78
Nation	Iranian	53 (86.9 %)	50 (80.6 %)	54 (90.0 %)	0.32
	Afghan	8 (13.1 %)	12 (19.4 %)	6 (10.0 %)	
Marital status	Single	24 (39.3 %)	27 (43.5 %)	25 (41.7 %)	0.89
	Married	37 (60.7 %)	35 (56.5 %)	35 (58.3 %)	
Living area	Urban	38 (62.3 %)	38 (61.3)	43 (71.7 %)	0.32
	Rural	23 (37.7 %)	24 (38.7 %)	17 (28.3 %)	
Education	Illiterate	10 (16.4 %)	14 (22.6 %)	9 (15.0 %)	0.62
	Under diploma	31 (50.8 %)	34 (54.8 %)	35 (58.3 %)	
	Diploma and academic	20 (32.8 %)	14 (22.6 %)	16 (26.7 %)	
Job	Manual	18 (29.5 %)	23 (37.1 %)	29 (48.3 %)	0.10
	Office-worker	43 (70.5)	39 (62.9 %)	31 (51.7 %)	

Tobacco use related factors did not differ significantly between the three groups. The mean number of cigarettes smoked daily was 15.30, 15.66 and 17.01 among the control group, brief advice group and combined intervention group respectively (*p* = 0.62). The rate of contemplation for quitting within the next month was found to be 42.6, 46.8 and 45 % for the same arrangement of groups (*p* = 0.89) and the mean score of motivation from 10, was calculated to be 5.57, 5.10 and 5.51 (*p* = 0.43), respectively (Table 2). Compliance with TB therapy was also found to be similar in the three groups.

**Table 2:** Smoking-related characteristics of the newly-diagnosed PTB patients recruited from Tehran, Iran during 2012–2013

Factor	Control	Brief advice	Combine intervention	*p*-value
Smoking initiation age	21.77 ± 7.43	22.26 ± 9.47	22.67 ± 9.90	0.94
Smoking status				
Daily	55 (90.2 %)	59 (95.2 %)	55 (91.7 %)	0.56
Occasional	6 (9.8 %)	3 (4.8 %)	5 (8.3 %)	
Smoking Cause				
Relaxation	7 (11.7 %)	6 (9.8 %)	2 (3.3 %)	0.47
Enjoyment	0 (0 %)	2 (3.3 %)	2 (3.3 %)	
Addiction	25 (41.7 %)	29 (47.5 %)	30 (50.0 %)	
Habitual	28 (46.7 %)	24 (39.3 %)	26 (43.0 %)	
Number of daily cigarettes	15.30 ± 10.45	15.66 ± 10.98	17.02 ± 10.87	0.62
PY^a^	20.02 ± 17.39	20.04 ± 20.58	22.11 ± 21.19	0.89
Fg^b^	6.43 ± 2.88	5.76 ± 2.47	6.71 ± 2.54	0.6
Hookah consumption				
Yes	15 (24.6 %)	14 (22.6 %)	16 (26.7 %)	0.38
No	46 (75.4 %)	48 (77.4 %)	44 (73.3 %)	
Previous quit attempts				
Yes	39 (63.9 %)	35 (56.5 %)	34 (56.7 %)	0.63
No	22 (36.1 %)	27 (43.5 %)	26 (43.3 %)	
Intention to quit				
Yes	40 (65.6 %)	42 (67.7 %)	34 (56.7 %)	0.41
No	21 (34.4 %)	20 (32.3 %)	26 (43.3 %)	
Intention to quit in next month	26 (42.6 %)	29 (46.8 %)	27 (45.0 %)	0.89
	35 (57.4 %)	33 (53.2 %)	33 (55 %)	
Quit motivation	6.31 ± 2.38	6.18 ± 2.71	6.58 ± 2.34	0.67
Quit insurance	5.57 ± 2.36	5.10 ± 2.47	5.51 ± 2.31	0.43
Pre intervention Pico	7.49 ± 2.23	7.17 ± 2.30	7.66 ± 2.19	0.53

^a^Pack/Year: calculated by multiplying the number of packs of cigarettes smoked per day by the number of years the subject has smoked

^b^Fagerstrom score: a scale for measuring nicotine dependency

**Fig. 2:**
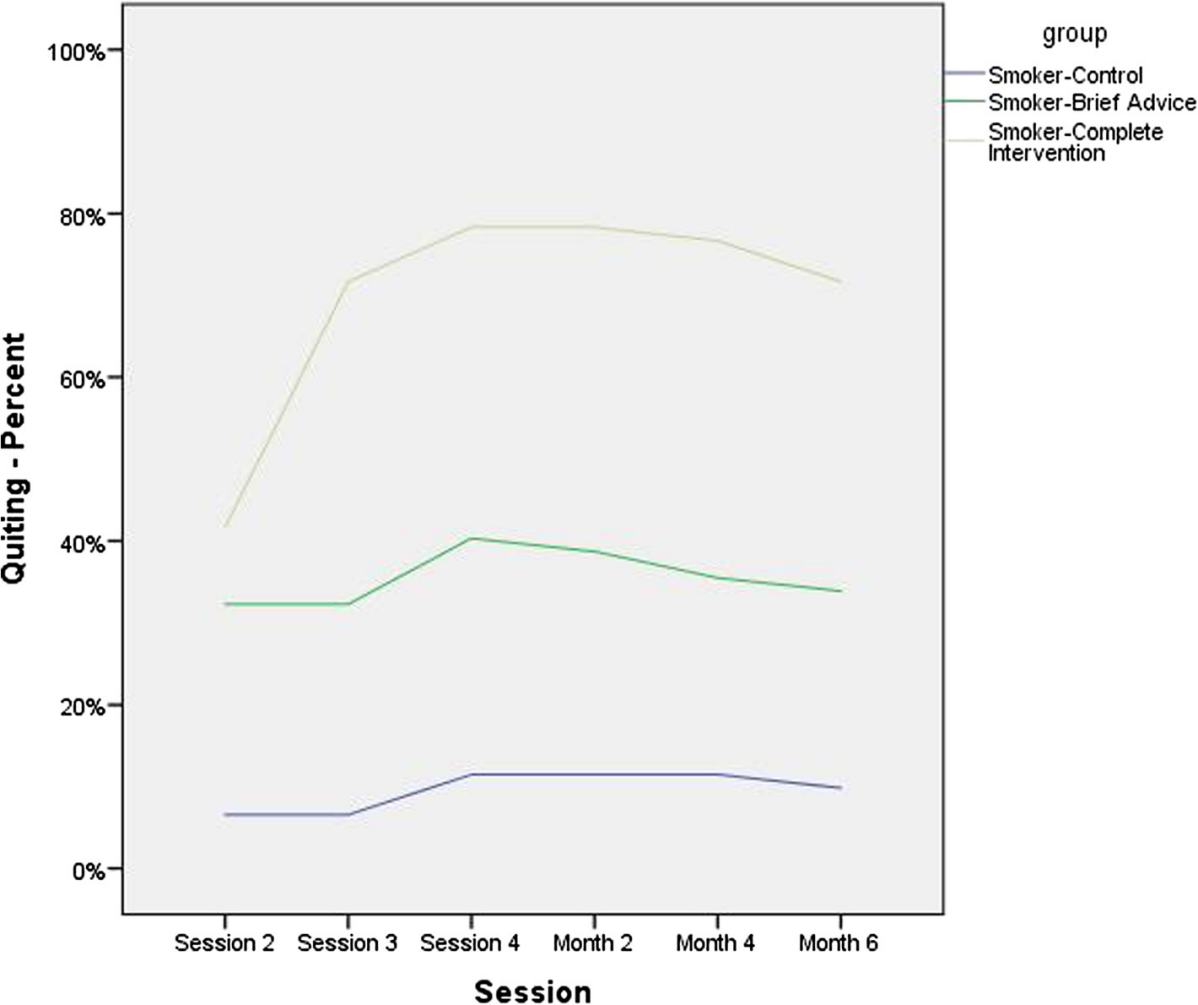
The trends of changes in smoking cessation status in the three groups of the newly-diagnosed PTB patients recruited from Tehran, Iran during 2012–2013

Considering the trend of changes in rate of smoking cessation from the third day through the end of the sixth month, combined intervention group was found to present with significantly higher figures (*p* < 0.001) (Fig. 2). Evaluation of continuous cessation at the end of the second month found a considerably higher rate in the combined intervention group with 78.3 % compared to the brief advice group with 38.7 % and the control group with 11.5 % (*p* < 0.001) (Table 3). Assessment after 6 months yielded similar results with 71.7 % for the combined intervention group, 33.9 % for the brief advice group and 9.8 % for the control group and the differences were found to be statistically significant (*p* < 0.001) (Table 3).

**Table 3:** The trend of changes in smoking cessation status in the three groups of the newly-diagnosed PTB patients recruited from Tehran, Iran during 2012–2013

Smoking status	Group			
	Control (61 cases)	Brief Advice (62 cases)	Combined intervention (60 cases)	*p*-value
	N (%)	N (%)	N (%)	
Session 2				
Quit	4 (6.6 %)	20 (32.3 %)	25 (41.7 %)	<0.001
Smoker	57 (93.4 %)	42 (67.7 %)	35 (58.3 %)	
Session 3				
Quit	4 (6.6 %)	20 (32.3 %)	43 (71.7 %)	<0.001
Smoker	57 (93.4 %)	42 (67.7 %)	17 (28.3 %)	
Session 4				
Quit	7 (11.5 %)	25 (40.3 %)	47 (78.3 %)	<0.001
Smoker	54 (88.5 %)	37 (59.7 %)	13 (21.7 %)	
Month 2				
Quit	7 (11.5 %)	24 (38.7 %)	47 (78.3 %)	<0.001
Smoker	54 (88.5 %)	38 (61.3 %)	13 (21.7 %)	
Month 4				
Quit	7 (11.5 %)	22 (35.5 %)	46 (76.7 %)	<0.001
Smoker	54 (88.5 %)	40 (64.5 %)	14 (23.3 %)	
Month 6				
Quit	6 (9.8 %)	21 (33.9 %)	43 (71.7 %)	<0.001
Smoker	55 (90.2 %)	41 (66.1 %)	17 (28.3 %)	

Since analyses (Chi-Square test for qualitative variables and Kruskal-Wallis for quantitative variables) performed on smoking-related variables including age of onset, smoking status, reason for smoking, number cigarettes per day, history of quitting, motivation for quitting, contemplation for quitting, pack year, and Fagerstrom score did not found any significant difference, they were excluded and only the variables of age, nationality, marital status, location, education and occupation were included in modeling.

Finally through GEE analysis, the rates of not being an active smoker at each time point were assessed in the three groups taking multiple observations at different times into account and controlling for confounding factors. The total quit differences were statistically significant in the groups (*p* < 0.001). As resulted, the patients in combined intervention group and brief advice group respectively had 35 times (*p* < 0.001, OR = 35.26, 95 % CI = 13.77–90.32) and 7 times (*p* < 0.001, OR = 7.14, 95 % CI = 2.72–18.72) more odds of not being an active smoker at each time point compared to the control group.

## Discussion

Tobacco use and tuberculosis have been described as an epidemic twisted skein. Tobacco use, is considered as one of the most important health risk factors in the countries in which smoking has become epidemic and along with tuberculosis, these health issues have become extensively prevalent in these regions [3].

In their mathematical model, Basu et al. estimated that a strict control on tobacco use leading to a 1 % annual decrease in prevalence of smoking can prevent 27 million smoking- and TB-related deaths till 2050. On the other hand, a 50 % rise of tobacco use among adult population (a phenomenon observed in countries with high prevalence of smoking) can add 34 million extra TB-related deaths till 2050 [29].

The results of the present survey showed that implementation of a combined intervention for newly-diagnosed smear positive pulmonary TB patients can lead to a significant increase (71.7 %) in the rate of continuous smoking cessation at the end of sixth months. Moreover, a brief advice intervention was also found to increase this rate to a considerable figure of 33.9 % compared to 9.8 % in the control group. Based on the logistic regression analysis carried out, these two interventions, compared to the control group, can increase the odds of not being an active smoker at each time point by 35 and 7 times, respectively. This figure, particularly for the combined intervention group, was significantly higher than the observations of Siddiqi et al. in Pakistan [24]. They showed that behavioral support in combination with bupropion increased the rate of continuous smoking cessation after 6 months to 45.4 %, compared to 41 % for behavioral support alone and 8.5 % for the control group. In their survey behavioral support comprised of two counseling sessions and bupropion was administered with a dose of 150 mg/d for seven weeks. Patients were evaluated after 1, 5 and 25 weeks. The different results might be due to the disagreements between the two surveys regarding their methods; 2 counseling sessions vs. 4, and 7 weeks of 150 mg/d bupropion vs. 9 weeks of 300 mg/d bupropion in our study. Also the evaluation checkpoints differed between the two studies. Moreover, they included the patients suspected to have TB while our study population comprised of TB patients with definite diagnoses and so our results can more appropriately be generalized to the whole population of TB patients being treated by DOTS course.

The study conducted by Awaisu et al. in Malaysia reported higher figures than ours. At their 6 month evaluation, 77.5 % of the patients in the intervention group (behavioral therapy combined with nicotine replacement therapy) and 8.7 % of the patients in the control group were found to be in a 4 month continuous smoking cessation. In their survey, new TB cases with positive or negative smear results who were motivated to quit within the next 30 days of TB diagnosis were assigned to the intervention group with 11 counseling sessions and patients in the stages of pre-contemplation and contemplation were put into the control group [23]. The fact that they included patients that are already willing to quit in the next 30 days might be the reason for their higher yielded efficacy.

Campbell et al. assessed the effectiveness of brief advice in TB patients in Nepal and found that 39 % of their intervention group managed to be smoke free in 6 months while none of their control subjects were able to stay clean in that period [30]. In the study conducted by Siddiquea et al. in Bangladesh, 82 % of the subjects declared that they had not smoked in 6 months and the rate of smoking cessation was reported to be higher among pulmonary TB patients compared to extra-pulmonary TB cases [25]. El sony et al. aimed to evaluate the effects of repeated brief advice interventions on smoking cessation among new pulmonary TB patients in Sudan. In their survey, 47 % of patients declared that they had not smoked in 6 months. However, they only assessed male subjects and no biochemical validation of smoking cessation was carried out [20].

Stead et al. evaluated 41 trials conducted on normal smoker populations and found that brief advice can significantly increase the rate of quitting (RR = 1.66). They also found complete intervention to be more effective than brief advice for this means (RR = 1.37) [31]. However, it should be noted that being diagnosed with TB and the patient being ill, can be enough on its own to motivate the patient to quit [32–34] and this can be the reason for the better results yielded from implementing smoking cessation interventions on TB patients.

In our study we included patients of both genders but as reported, the majority of our study population was male. Although, the results can still be generalized to the whole population since the proportion of male to female in our sample population is quite similar to the figure reported for the smokers in the whole Iranian population [35] and the considerably lower prevalence of smoking among females led to inclusion of mostly male subjects in this study.

The discrepancies observed between the mentioned studies can be attributed to the variabilities in the design of the surveys, their study populations, the outcomes measured and the time at which they are evaluated. However, all these studies showed significant improvements in smoking cessation rates after various cessation programs and interventions.

As mentioned, tobacco use can increase the prevalence of TB and its related mortalities and so strict control over smoking can prevent millions of TB related deaths. Accordingly, the need for inclusion of smoking cessation measures in TB control programs seems undeniable. This survey paved the way for further investigations on this matter and provided evidence supporting the need to establish smoking cessation clinics in TB control health centers.

One of the limitations of this study was that we only measured the outcome of smoking cessation in these patients until the end of TB treatment, while previous studies have shown that many patients start smoking again after completing their TB therapy [15]. This might over-estimate the effects of evaluated cessation measures in quitting since patients tend to take smoking cessation more seriously when it is concurrent with their TB treatments and although the adverse effects of smoking and the utmost importance of complete continuous cessation are thoroughly explained to them, they might ignore these facts and start smoking again. Moreover, it has been shown that smoking is a risk factor for relapse of TB disease in patients with successful treatments which increases the costs and imposes additional burdens to the public health systems. Therefore, further investigations are required to assess the long-term outcomes of these cessation programs even particularly after completion of TB treatments.

Furthermore, we used the expiratory carbon monoxide concentration to confirm smoking status of the subjects which is not an absolute indicator of smoking status. So it is recommended that future studies incorporate urine cotinine and nicotine derivatives as their outcome measures to yield more reliable results.

## Conclusion

Putting it all together, regarding the smoking related morbidities and mortalities of TB patients and considering the significant effects of educational interventions provided as combined method or brief advice, it seems logical to include these measures as a part of TB control programs.

## Abbreviations

AIDS, acquired immunodeficiency syndrome; CD, cluster of differentiation; GEE, Generalized Estimating Equations; HIV, human immunodeficiency virus; QIC, Quasi-Akaike Information Criterion; TB, tuberculosis; WHO, World Health Organization
